# Relationships between rumen methanogens and fungal communities and their response to changes in alfalfa forms and starch in sheep diets

**DOI:** 10.3389/frmbi.2025.1567462

**Published:** 2025-04-09

**Authors:** Wenliang Guo, Meila Na, Shuwei Liu, Kenan Li, Haidong Du, Jing Zhang, Yu Zhang, Renhua Na, Yulan Liu

**Affiliations:** ^1^ College of Animal Science, Inner Mongolia Agricultural University, Hohhot, China; ^2^ Forage Processing and Animal Nutrition Research Center, Grassland Research Institute of Chinese Academy of Agricultural Sciences, Hohhot, China; ^3^ Department of Technology, Inner Mongolia Zhamuqin Agriculture and Animal Husbandry Technology Co., Ltd, Ulanhot, China

**Keywords:** alfalfa hay, alfalfa silage, rumen degradable starch, rumen, methanogens, fungi

## Abstract

Alfalfa forms and rumen degradable starch (RDS) levels in diets can profoundly affect growth performance and rumen fermentation patterns, this influence may result in variations in rumen microbiota. However, the effects of RDS levels on methanogenic and fungal communities in alfalfa hay (AH) or alfalfa silage (AS) diets, and the interaction between methanogens and fungi with growth performance and rumen fermentation patterns, remain unknown. In this study, a 2 × 2 factorial design resulted in four diets: two alfalfa forms (AH and AS) and two RDS levels (LR: 14.85% DM RDS; and HR: 20.21% DM RDS). We used 32 female Suffolk sheep for the experiment. On day 75 (including a 15-day transition period and a 60-day trial period), rumen content was collected after slaughter to examine the ruminal methanogens and fungi. The AHHR diet reduced the methanogen Chao 1 index compared to the AS diets (*P* < 0.05), and the Shannon index was lower than in the ASLR diet (*P* < 0.05). The fungi Chao 1 index was higher in the AH diets than in the ASHR diet (*P* < 0.05), and the fungi Shannon index was higher in the LR diets than in the HR diets (*P* < 0.05). The relative abundance of *Aspergillus* in the AHLR diet was significantly higher than in the AS diets (*P* < 0.01), and the relative abundance of *Occultifur* and *Meyerozyma* were decreased in the AH diets than in the AS diets (*P* < 0.05). The LEfSe analysis showed that *Methanobrevibacter_sp_YE315* and *Methanobrevibacter_sp_AbM4* were enriched in the ASLR diet, while *Methanobrevibacter_millerae* was enriched in the ASHR diet. For the fungal biomarkers, the AHLR diet included *Aspergillus*, *Metschnikowia*, and *unclassified_f:Stachybotryaceae*; the AHHR diet included *stachybotrys*, *Stemphylium*, and *Cystobasidium*; the ASLR diet included *unclassified_k:Fungi*, *Trichothecium*, and *Psathyrella*; and the ASHR diet included *Alfaria*. The correlation analysis results showed the relative abundance of *Methanobrevibacter*, *Methanoculleus*, *Penicillium*, *Cladosporium*, and *Exophiala* and the concentrations of isobutyrate and isovalerate, which may provide deeper insights into the previously observed differences.

## Introduction

Alfalfa is an important roughage for ruminant feed due to its excellent amino acid profile and low lignocellulose content. Haymaking and ensiling are the most frequent forms of processing alfalfa (Wayne et al., 2020; Radovic et al., 2009; Sun et al., 2023). Ensiling is an anaerobic microbial-based fermentation process dominated by lactic acid bacteria, which produce lactic acid and volatile fatty acids (VFAs) necessary for pH decline and the inhibition of harmful microorganisms (Jiang et al., 2020). Furthermore, environmental conditions that develop during ensiling favor the proliferation of the phylum *Firmicutes* (Yang et al., 2020). This leads to variations in the microbiota of alfalfa silages (AS) compared to alfalfa hay (AH), and these changes further alter the microbial community in the rumen. For instance, silage treatment disrupts the structure of plant lignocellulosic materials through anaerobic fungi. The abundance of cellulolytic microorganisms was increased after feeding a fermented diet (He et al., 2023), and the gastrointestinal microbial community undergoes changes (Xu et al., 2023). Our previous studies have shown that AS diets increased amino acid degradation and the abundance of associated bacteria in the rumen of sheep (Guo et al., 2025), implying that AS had the potential to alter rumen microbiota.

Livestock contributes approximately 14.5% of global greenhouse gas (GHG) emissions, with enteric methane (CH_4_) emissions accounting for up to 40% of livestock**’**s GHG emissions (Eugène et al., 2021). CH_4_ is one of the fermentation products produced by archaea utilizing carbon dioxide (CO_2_) and hydrogen (H_2_), accounting for approximately 2%–12% of the host**’**s ingested feed energy lost. Nearly all the archaea identified are methanogens known to be resident in the rumen (Johnson and Johnson, 1995), and the abundance of methanogens has a positive correlation with methane emissions (Wallace et al., 2015; Niu et al., 2020). Current research shows that the effect of feeding AH or AS on ruminal methane emissions in ruminants appears to be slight. Gislon et al. (2020) demonstrated that methane production per unit of feed intake and milk production was not different in cows fed AH or AS and in the case of dairy goats (Fernández et al., 2019). *In vitro* tests have found similar results. Getachew et al. (2004) found no difference in gas production between AH and AS using *in vitro* techniques. Xue et al. (2020) evaluated *in vitro* rumen methane production responses to different forage ratios of AH and AS. The results indicate that methane production at 48 h was greater for silages compared to hays. However, the above experiments did not determine the methanogens.

Rumen fungi are considered to play key roles in the degradation of plant lignocellulosic materials. The average abundances indicate that fungi represent 10% to 20% of the rumen microbiota (Elekwachi et al., 2017). After ensiling, most aerobic fungi genera were killed and/or inhibited, and only a few anaerobic fungi genera are present along with lactic acid bacteria to secrete extracellular enzymes to degrade cell walls (Jiang et al., 2020; Jia et al., 2024). Microbial-rich silage feeding is generally considered to alter the rumen microbial community (Akin et al., 1988). Although many studies have examined the effects on rumen fermentation parameters (Hristov et al., 2001; Beauchemin et al., 1997) and bacterial community (Guo et al., 2025) in AH or AS diets, the effects on fungi were unknown.

Starch is a major component of cereals and the primary energy source for the fattening of ruminants. Corn and wheat have been important diet sources for ruminant and non-ruminant animals due to their high production yields and high starch content (Li et al., 2019; Ma et al., 2022). Wheat, a cereal with a high rumen degradable starch (RDS) content, is commonly included in the diets of Australian dairy cows (Moate et al., 2017; Shen et al., 2020; Zhang et al., 2024). The starch degradation rate of wheat is 24.8% higher than that of corn (Ferraretto et al., 2013). Studies have shown that increasing dietary RDS improves feed efficiency (Jin et al., 2023; Karim et al., 2008), optimizes the digestion of carbohydrates and protein (Moate et al., 2017; Gao et al., 2024), reduces methane emissions (Savin et al., 2022), and increases protein flow to the small intestine (Zhang et al., 2024; Plascencia et al., 2018). In addition, increased dietary RDS alters the diversity and abundance of rumen bacteria (Jiang et al., 2020; Guo et al., 2025a; Li et al., 2024). However, analyses of methanogens and fungi were still lacking in these experiments. Methanogens and fungi have a certain symbiotic relationship, and fungal degradation fiber provides an H_2_ substrate for methanogens, which promotes their reproduction. Therefore, a joint analysis of methanogens and fungi may provide a better understanding of the effects of phenotypic variables. We hypothesized that dietary alfalfa forms and RDS levels will result in different rumen methanogens and fungi communities in sheep, and that these microbial differences will explain the previously observed differences in growth performance and rumen fermentation.

## Materials and methods

### Animals, diets, and experimental d esign

All animal procedures were conducted according to protocols approved by the Animal Welfare and Ethics Committee of Inner Mongolia Agricultural University (NND2024053). This study was carried out at the Experimental Farm of Inner Mongolia Agricultural University, located in Tumurt Left Banner, Hohhot, China. As part of a previous study (Guo et al., 2025a), a total of 32 female Suffolk sheep with similar weights (initial weight 27.28 ± 3.4 kg) and aged 3 months were randomly assigned to four dietary treatments in a completely randomized design (n = 8 per treatment). Four diets were designed with 2 × 2 factors: two alfalfa forms (AH: alfalfa hay; AS: alfalfa silage) and two RDS levels (LR: 14.85%DM RDS; and HR: 20.21%DM RDS). Alfalfa hay (AH) and alfalfa silage (AS) were determined based on the results of Ainslie et al. (2014). The RDS level was determined based on the results of Guo et al. (2025b). The treatment diets were formulated to be isocaloric and isonitrogenous and met the NYT816-2021 recommendations (**Tables 1**, **2**). Sheep were housed in an outdoor rearing system in individual pens (1.0 × 1.0 m^2^) bedded with sand. The experimental period was 75 days, including a 15-day transition period and a 60-day trial period. Sheep were fed twice daily at 09:00 and 16:00 as Total Mixed Ration (TMR) for *ad libitum* intake. Rumen contents were collected after slaughter and stored at −80°C to examine the ruminal methanogens and fungi.

**Table 1:** The nutritive values of feed ingredients (g/kg dry matter).

Nutrient^1^	Corn stalk	Alfalfa hay	Alfalfa silage	Corn	Wheat	Soybean meal	Wheat bran
Dry matter	94.44	91.70	37.40	86.90	85.70	91.20	90.2
Digestible energy, MJ/kg	9.58	10.96	10.77	14.86	14.91	16.41	12.81
Crude protein	4.52	18.30	19.10	8.50	13.50	47.60	17.4
Starch	3.5	–	–	70.40	63.10	–	–
Acid detergent fiber	42.92	30.75	26.58	3.60	4.20	10.10	13.80
Neutral detergent fiber	71.32	46.67	36.67	9.80	12.50	19.60	40.12
Ether extract	3.35	2.50	3.50	3.84	1.98	7.12	4.39
RDS	–	–	–	38.08	49.71	–	–
RDP	–	43.00	58.00	–	–	–	–

^1^RDS, rumen degradable starch; RDP, rumen degradable protein.

**Table 2:** Ingredient compositions and nutritive values of experimental feeds (g/kg dry matter).

Ingredient	Treatment ^1^				Nutrient^3^	Treatment			
	AH		AS			AH		AS	
	LR	HR	LR	LR		LR	HR	LR	HR
Corn stalk	10	10	10	10	Dry matter	87.75	86.28	66.03	64.56
Alfalfa hay	40	40	0	0	Digestible energy, MJ/kg	12.18	12.18	12.14	12.14
Alfalfa silage	0	0	40	40	Crude protein	13.93	13.84	14.33	14.24
Corn	39	10	39	10	starch	28.15	28.40	29.87	30.12
wheat	0	33	0	33	Acid detergent fiber	20.57	20.37	20.33	20.13
Soybean meal	5	1	5	1	Neutral detergent fiber	30.50	30.60	29.34	29.44
Wheat bran	3.75	2.75	3.75	2.75	Ether extract	3.72	3.71	4.12	4.11
Soybean oil	0.25	1.25	0.25	1.25	RDS	14.85	20.21	14.85	20.21
Calcium hydrogen Phosphate	0.25	0.25	0.25	0.25	RDP	2.94	2.94	4.38	4.38
limestone	0.75	0.75	0.75	0.75	RDS/RDP	5.05	6.87	3.39	4.61
Salt	0.5	0.5	0.5	0.5					
Premix ^2^	0.5	0.5	0.5	0.5					

^1^AHLR, alfalfa hay and low (14.85% DM) RDS; AHHR, alfalfa hay and high (20.21% DM) RDS; ASLR, alfalfa silage and low (14.85% DM) RDS; ASHR, alfalfa silage and high (20.21% DM) RDS. ^2^The premix contained/kg diet: vitamin A 6 000 IU, vitamin D3 2000 IU, vitamin E 15 IU, vitamin K3 1.8 mg, vitamin B1 0.35 mg, vitamin B2 8.5 mg, vitamin B6 0.9 mg, vitamin B12 0.03 mg, D-pantothenic acid 16 mg, nicotinic acid 22 mg, folic acid 1.5 mg, biotin 0.15 mg, Cu 8 g, Fe 40 mg, Mn 20 mg, Zn 40 mg, I 0.8 mg, Se 0.3 mg, Co 0.3 mg. ^3^RDS, rumen-degradable starch; RDP, rumen-degradable protein.

### Rumen bacterial DNA extraction and analysis

Metagenomic DNA was extracted from each rumen sample using the E.Z.N.A.**^®^** soil DNA Kit (Omega Biotek, Norcross, GA, USA), following the manufacturer**’**s instructions. The hypervariable V4 region of the methanogens**’** 16S rRNA gene and the fungal internal transcribed spacer were amplified by PCR using a T100 Thermal Cycler. The methanogens primers used in the current study were MLfF-F: 5´-GGTGGTGTMGGATTCACACARTAYGCWACAGC-3´ and MLfF-R:5´-TTCATTGCRTAGTTWGGRTAGTT-3´. The fungal primers used in the current study were ITS1-1F-F: 5′-CTTGGTCATTTAGAGGAAGTAA-3′ and ITS1-1F-R: 5′-GCTGCGTTCTTCATCGATGC-3′. The amplification system (20 μL) was as follows: 4 µL 5×FastPfu Buffer, 2 µL 2.5 mmol/L dNTPs, 0.4 µL forward primers (5 mmol/L), 0.8 µL reverse primers (5 mmol/L), 0.2 µL BSA, template DNA 10 ng, and make up ddH2O to 20 µL. The steps of PCR amplification were as described previously (Guo et al., 2025a). PCR products were recovered by gel extraction in AquaPōr LM low-melt agarose (National Diagnostics, Atlanta, GA) using the Zymoclean Gel DNA Recovery Kit (Zymo Research, Irvine, CA). After the constructed library was quantified by Qubit and real-time PCR, sequencing was performed using the Illumina NovaSeq 6,000 sequencing platform.

The amplicon sequences were quality-controlled and merged by FASTP (version 0.19.6) and FLASH (version 1.2.7), respectively. Briefly, amplicon sequence denoising, merging, and chimeric sequence removal were conducted as described previously (Li et al., 2024) using the DADA2 plugin at a 97% sequence similarity threshold in Uparse software. Bioinformatic analysis of the rumen methanogens and fungi was carried out using the Majorbio Cloud platform (https://cloud.majorbio.com, accessed on 28 September 2024). QIIME2 software (v.1.8.0) was used to assess alpha diversity measurements including Chao 1 and Shannon indices, with significant differences analyzed using the Wilcox rank sum test. QIIME2 software assessed beta diversity measurements including principal coordinates analysis (PCoA) based on Bray–Curtis dissimilarities and relative abundance, with significant differences analyzed using analysis of similarity (ANOSIM). The software Linear discriminant analysis Effect Size (LEfSe) (Version 1.0) was used to analyze the effects of different diet treatments of rumen methanogens and fungi. Only microbial communities**’** linear discriminant analysis (LDA) score values greater than 3.0 were identified as specific microbiota unique to the diet treatments. Spearman**’**s rank correlation analyzed the relationship between the rumen methanogens and the top 10 fungi at the genus level, with a coefficient of > |0.4|, *P* < 0.05 considered significant. The rumen methanogens and fungal sequencing data of this study are available in the NCBI SRA database with the BioProject ID: PRJNA1236662.

### Data processing and analysis

The methanogen and fungi data were analyzed using one-way ANOVA using statistical analysis software SAS (Version 9.2, SAS Institute Inc. Cary, NC, USA). Duncan**’**s multiple range test (DMRT) was conducted to evaluate the differences among the treatments, along with the mixed model procedure in SAS. The model included alfalfa forms (AS: AH), RDS levels (LR: HR), and the two-way interaction between alfalfa forms and RDS levels, and was considered significant at P < 0.05 and extremely significant at P < 0.01. Data were presented as averages.

Methanogen relative abundance, fungal relative abundance, and the relationship between methanogens and fungi in relation to growth performance and rumen fermentation parameters were assessed using Spearman**’**s rank correlation, with a coefficient of > |0.4|, p < 0.05 considered significant.

## Results

### Methanogen community composition

**Figure 1:**
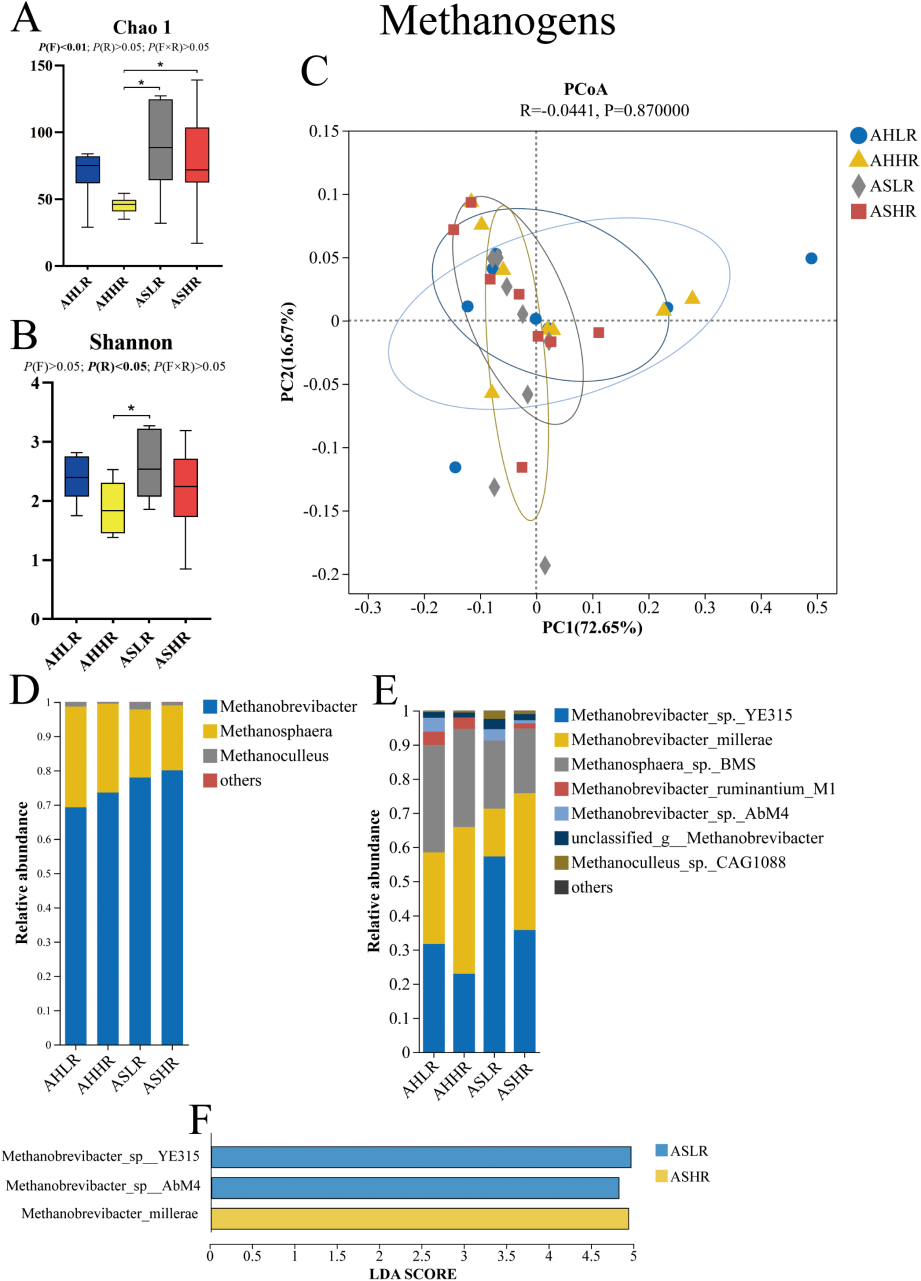
Dietary alfalfa forms and RDS levels altered methanogen communities in the rumen of sheep. **(A)** Chao 1 index of alpha diversity under genus level. **(B)** Shannon index of alpha diversity under genus level. **(C)** Principal coordinates analysis (PCoA) at the genus level. **(D)** Methanogens taxa averaged at the genus level. **(E)** Methanogen taxa averaged at the species level. **(F)** LEfSe analysis of methanogen at the species level for different treatments. AHLR, alfalfa hay and low (14.85% DM) RDS; AHHR, alfalfa hay and high (20.21% DM) RDS; ASLR, alfalfa silage and low (14.85% DM) RDS; ASHR, alfalfa silage and high (20.21% DM) RDS. * means a significant difference *P* < 0.05. *P* (F) = alfalfa hay versus alfalfa silage (AH vs. AS); *P* (R) = low (14.85% DM) RDS versus high (20.21% DM) RDS; *P* (F×R) = alfalfa forms by RDS levels interaction.

For our methanogen alpha diversity analysis, the Chao 1 index was higher in the AS diets compared to the AHHR diet (*P* < 0.05). Similarly, the Shannon index was higher in the ASLR diet compared to the AHHR diet (*P* < 0.05) (**Figures 1A, B**). These results suggest that there were more lowly abundant methanogen genera in the AS diets and a co-occurrence of highly abundant methanogen genera in the ASLR diet. However, beta diversity analysis revealed that the Bray–Curtis dissimilarities in the methanogen communities were similar among the four diets (**Figure 1C**).

All methanogens were identified as belonging to the *Euryarchaeota* phylum, including three genera of *Euryarchaeota* present in all of the samples, with *Methanobrevibacter* being more prevalent (69.25%–80.05%) than *Methanosphaera* (18.96%–29.42%) and *Methanoculleus* (0.5%–2.11%). There was no significant difference in the relative abundance of these three genera among the four diets (*P* > 0.05) (**Figure 1D**; **Table 3**). At the species level, *Methanobrevibacter_sp._YE315* (31.68%–57.25%), *Methanobrevibacter_millerae* (14.02%–26.78%), and *Methanosphaera_sp._BMS* (19.9%–35.77%) dominated the methanogen communities, accounting for at least 90% of the methanogen species present (**Figure 1E**). LEfSe analysis results showed that *Methanobrevibacter_sp_YE315* and *Methanobrevibacter_sp_AbM4* were enriched in the ASLR diet, while *Methanobrevibacter_millerae* was higher in the ASHR diet (**Figure 1F**). There was no enrichment of methanogen in AH diets.

**Table 3:** Effect of dietary alfalfa forms and RDS levels on rumen relative abundance of methanogens and fungi at the genus level in sheep.

Item	Treatments ^1^				SEM	*P*-Value			
	AH		AS			Diet	Alfalfa	RDS	Alfalfa×RDS
	LR	HR	LR	HR					
Methanogens									
Methanobrevibacter	69.25	73.55	77.94	80.05	6.04	0.615	0.228	0.607	0.860
Methanosphaera	29.42	25.93	19.88	18.96	6.08	0.589	0.193	0.724	0.837
Methanoculleus	1.31	0.50	2.11	0.92	0.77	0.517	0.441	0.208	0.811
Fungi									
Candida	30.48	29.92	31.24	43.31	5.09	0.192	0.163	0.253	0.211
Sarocladium	16.62	26.69	19.84	16.62	4.15	0.276	0.408	0.409	0.114
Penicillium	11.42	8.57	13.84	11.00	1.62	0.148	0.130	0.078	0.999
Cladosporium	2.66	4.17	4.09	4.93	0.97	0.430	0.269	0.236	0.736
Aspergillus	11.30a	2.48ab	0.45b	0.57b	2.65	0.005	0.008	0.061	0.054
Acremonium	3.01	3.54	4.19	2.89	0.66	0.509	0.695	0.573	0.180
Exophiala	2.53	4.08	3.14	2.80	0.83	0.590	0.690	0.475	0.271
Occultifur	1.85	2.54	3.17	3.59	0.56	0.136	0.036	0.309	0.801
Meyerozyma	1.59	0.72	2.59	1.62	0.49	0.057	0.046	0.053	0.915
Fusarium	1.23	1.62	0.94	0.99	0.35	0.508	0.197	0.537	0.629
Hannaella	0.99	1.25	1.19	0.76	0.39	0.825	0.733	0.832	0.398
Wickerhamomyces	1.02	0.94	1.51	0.58	0.33	0.270	0.843	0.134	0.201
Gibellulopsis	0.82	0.76	1.15	1.05	0.22	0.558	0.171	0.725	0.924
Metschnikowia	1.72	0.23	0.88	0.87	0.44	0.126	0.821	0.088	0.091
Papiliotrema	0.72	1.12	0.82	0.99	0.29	0.788	0.958	0.349	0.705
Clavispora	0.36	1.33	0.70	0.17	0.65	0.788	0.544	0.737	0.267
Chrysosporium	1.91	0.10	0.01	0.01	0.93	0.406	0.296	0.339	0.343

^1^AHLR, alfalfa hay and low (14.85% DM) RDS; AHHR, alfalfa hay and high (20.21% DM) RDS; ASLR, alfalfa silage and low (14.85% DM) RDS; ASHR, alfalfa silage and high (20.21% DM) RDS. ^a, b^Significant differences within a row with different superscripts (P < 0.05). P (Diet) = four dietary treatments. P (Alfalfa) = alfalfa hay versus alfalfa silage (AH vs. AS); P (RDS) = low (14.85% DM) RDS versus high (20.21% DM) RDS (LR vs. HR); P (Alfalfa×RDS) = alfalfa forms by RDS levels interaction.

### Fungal community composition

**Figure 2:**
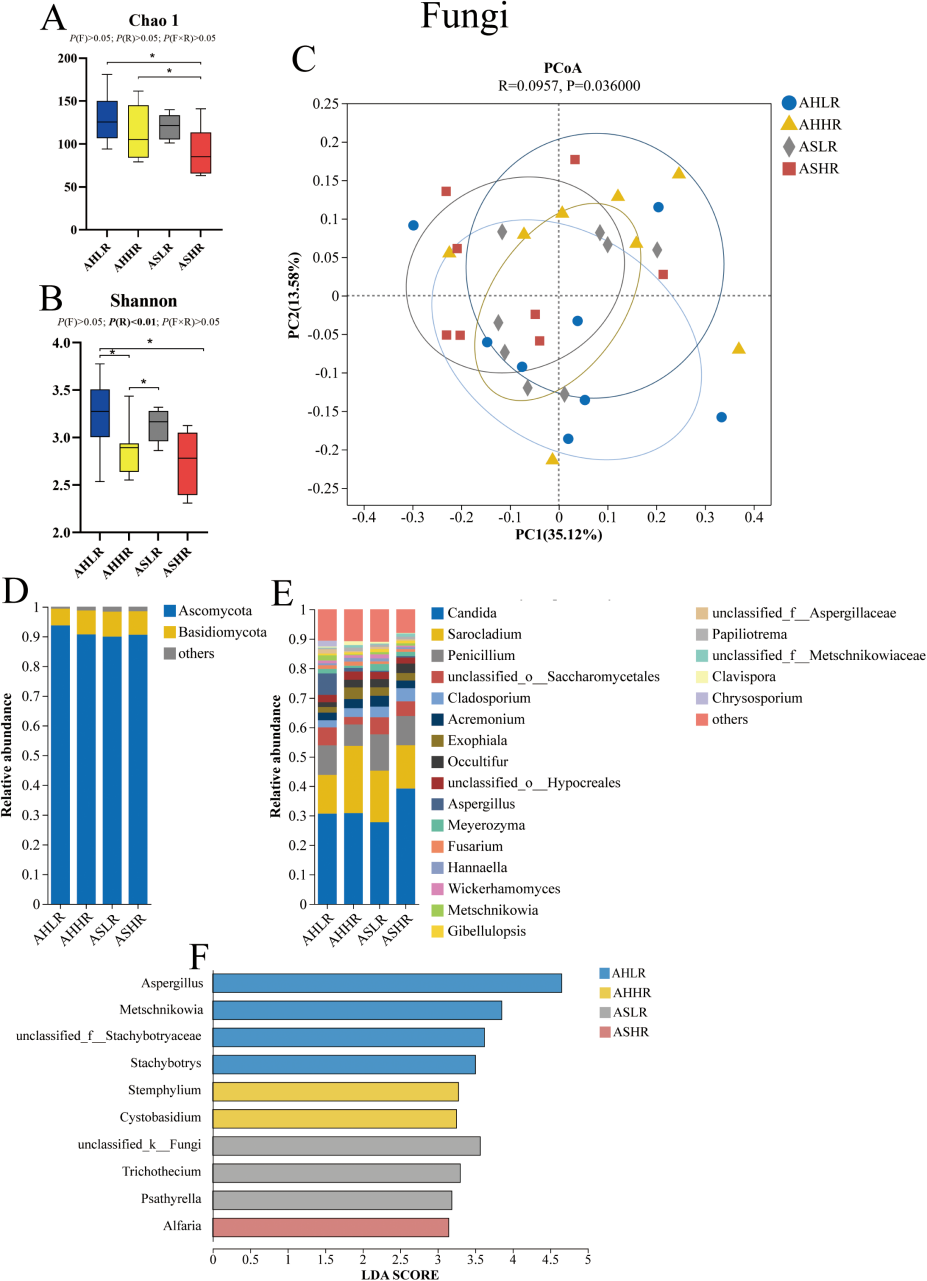
Dietary alfalfa forms and RDS levels altered rumen fungal communities in the rumen of sheep. **(A)** Chao 1 index of alpha diversity at the genus level. **(B)** Shannon index of alpha diversity at the genus level. **(C)** Principal coordinates analysis (PCoA) at the genus level. **(D)** Fungi taxa averaged at the phylum level. **(E)** Fungi taxa averaged at the genus level. **(F)** LEfSe analysis of fungi at the genus level for different treatments. AHLR, alfalfa hay and low (14.85% DM) RDS; AHHR, alfalfa hay and high (20.21% DM) RDS; ASLR, alfalfa silage and low (14.85% DM) RDS; ASHR, alfalfa silage and high (20.21% DM) RDS. * means a significant difference P < 0.05. *P* (F) = alfalfa hay versus alfalfa silage (AH vs. AS); P (R) = low (14.85% DM) RDS versus high (20.21% DM)RDS; P (F×R) = alfalfa forms by RDS levels interaction.

In our fungal alpha diversity analysis, the Chao 1 index was higher in the AH diets compared to the ASHR diet (*P* < 0.05). Additionally, the Shannon index was higher in the AHLR diet compared to the HR diets (*P* < 0.05). Furthermore, the Shannon index was also higher in the ASLR diet compared to the AHHR (*P* < 0.05) (**Figures 2A, B**). These results suggest that the AS diets contained a greater number of lowly abundant fungal genera compared to the ASHR diet, while the LR diets exhibited a higher diversity and co-occurrence of highly abundant fungal genera. However, the beta diversity analysis revealed that Bray–Curtis dissimilarities in the fungal community were similar among the four diets (**Figure 2C**).

The *Ascomycota* (89.95%–93.73%) and *Basidiomycota* (5.63%–8.42%) were the dominant phyla of fungi across all diets. These included five genera: *Candida* (29.92%–43.31%), *Sarocladium* (16.62%–26.69%), *Penicillium* (8.57%–13.84%), *Cladosporium* (2.66%–4.93%), and *Aspergillus* (0.45%–11.30%), which accounted for at least 70% of the fungi genera present (**Figures 2D, E**). The relative abundance of *Aspergillus* was higher in the AHLR diet than in the AS diets (*P* < 0.01). In contrast, the relative abundance of *Occultifur* and *Meyerozyma* were decreased in the AH diet compared to the AS diets (*P* < 0.05) (**Table 3**). Additionally, LEfSe analysis revealed four (*Aspergillus*, *Metschnikowia*, *unclassified_f:Stachybotryaceae*, and *stachybotrys*), two (*Stemphylium* and *Cystobasidium*), three (*unclassified_k:Fungi*, *Trichothecium*, and *Psathyrella*), and one (*Alfaria*) fungi taxa significantly associated with AHLR, AHHR, ASLR, and ASHR diets, respectively (**Figure 2F**).

### Correlation analyses

**Figure 3:**
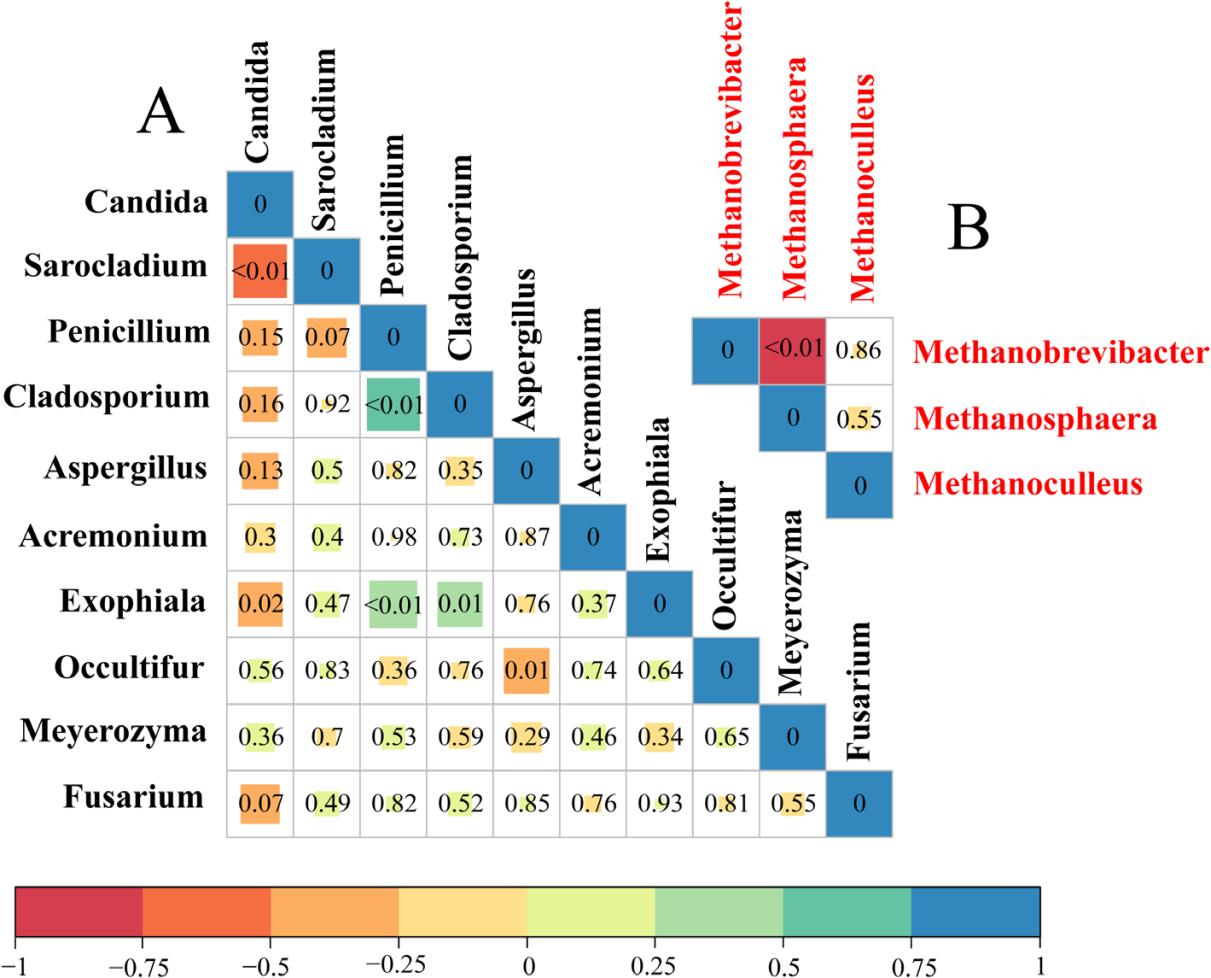
The correlations among the relative abundance of methanogen genera (red) and among the relative abundance of fungal genera (black) in different treatment diets. **(A)** Triangular heatmap of fungi. **(B)** Triangular heatmap of methanogens. The correlation is shown by Spearman’s rank correlation, ranging from −1 to 1, and represented by red (perfect negative correlation) to blue (perfect positive correlation).

The correlations within methanogens at the genus level were analyzed using triangular heatmaps, which revealed that the relative abundance of *Methanobrevibacter* was negatively correlated with *Methanosphaera* (*P* < 0.01). Among the top 10 fungi at the genus level, *Candida* showed a negative correlation with *Sarocladium* (*P* < 0.01) and *Exophiala* (*P* < 0.05). Furthermore, *Penicillium*, *Cladosporium*, and *Exophiala* showed positive correlations among themselves (*P* < 0.01). *Aspergillus* was also negatively associated with *Occultifur* (*P* < 0.01) (**Figure 3**).

**Figure 4:**
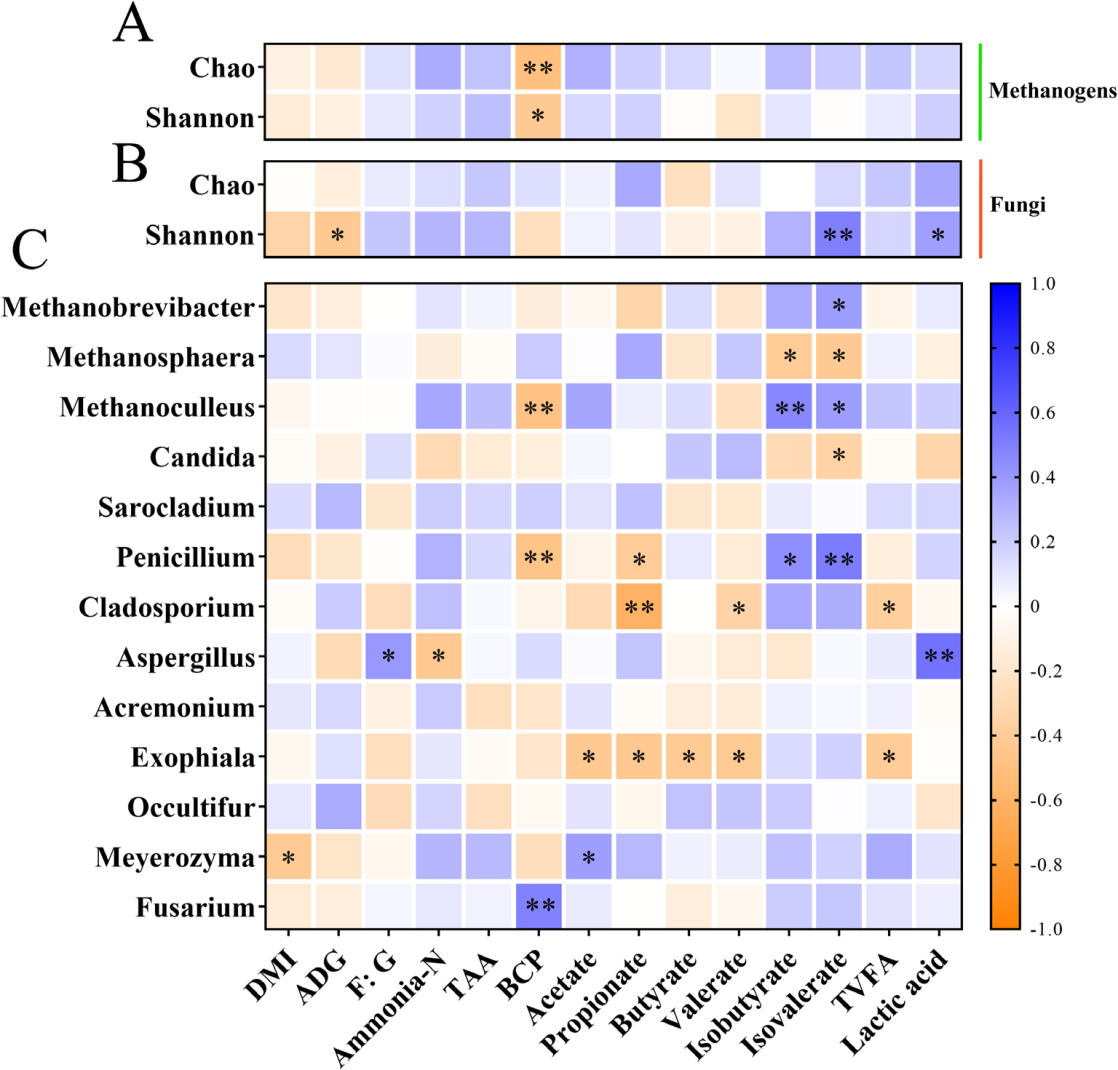
The relationship between methanogens and fungi and growth performance and rumen fermentation parameters. **(A)** Heat map of the relationship between the methanogen alpha diversity index and growth performance and rumen fermentation parameters. **(B)** Heat map of the relationship between the fungal alpha diversity and growth performance and rumen fermentation parameters. **(C)** Heat map of the relationship between the relative abundances of methanogens and fungi at the genus level and growth performance and rumen fermentation parameters. * and ** indicate significant differences at P < 0.05 and P < 0.01, respectively. ADG, average daily gain; DMI, daily matter intake; F:G, feed to gain ratio; TAA, total amino acid; BCP, bacterial protein; TVFA, total volatile fatty acids.

Heatmap correlation analysis was employed to determine correlations between the relative abundance of microorganisms (methanogens and top 10 fungal genera) and growth performance and rumen fermentation parameters (**Supplementary Material**, **Supplementary Table S1**; Guo et al., 2025a; **Figure 4**). At the alpha diversity level, the Chao 1 index (P < 0.01) and the Shannon index (*P* < 0.05) of methanogens were negatively correlated with bacterial protein (BCP), respectively. Furthermore, the fungal Shannon index displayed a negative correlation with average daily gain (ADG) (*P* < 0.05) and positive correlation with isovalerate (*P* < 0.01) and lactic acid (*P* < 0.05). At the methanogen genus level, the relative abundance of *Methanobrevibacter* showed a positive correlation with isovalerate (*P* < 0.05). *Methanosphaera* was negatively correlated with isobutyrate and isovalerate (*P* < 0.05). Moreover, *Methanoculleus* exhibited a negative correlation with BCP (*P* < 0.01) and positive correlations with isobutyrate (*P* < 0.01) and isovalerate (*P* < 0.05). At the fungal genus level, *Candida* was negatively correlated with isovalerate (*P* < 0.05). *Penicillium* displayed negative correlations with BCP (*P* < 0.05) and propionate (*P* < 0.01) while showing positive correlations with isobutyrate (*P* < 0.05) and isovalerate (*P* < 0.01). *Cladosporium* exhibited negative correlations with propionate (*P* < 0.01), valerate, and total volatile fatty acids (TVFA) (*P* < 0.05). *Aspergillus* was positively correlated with F: G (*P* < 0.05) and lactic acid (*P* < 0.01) while showing a negative correlation with NH_3_-N (*P* < 0.05). *Exophiala* demonstrated negative correlations with acetate, propionate, butyrate, valerate, and TVFA (*P* < 0.01). *Meyerozyma* was negatively correlated with daily matter intake (DMI) (*P* < 0.05) and positively correlated with acetate (*P* < 0.01). In addition, *Fusarium* had a negative association with BCP (*P* < 0.01).

## Discussion

Dietary forage and concentrate are the most important factors influencing growth performance, mainly by affecting rumen fermentation parameters and microbial community. In our previous study, all four diets altered rumen fermentation parameters and bacterial communities, yet only the HR diets improved growth performance. Therefore, understanding the effects of dietary alfalfa forms and RDS levels on rumen methanogens and fungal communities can aid in identifying differences in growth performance and rumen fermentation parameters. In this study, the Chao 1 and Shannon indices of methanogens in the AHHR diet were lower compared to those in the ASLR diet, and similarly, the Shannon index for fungi was also lower in the AHHR diet. The diversity of rumen methanogens and fungi in sheep was significantly different among the four diet treatments. Additionally, we identified taxa of methanogens and fungi that might be associated with rumen VFAs.

Archaea account for 2%–4% of rumen microbes, with 98% of them being methanogens (Li et al., 2024). *Methanobacteria* produce CH_4_ by regulating the partial pressure of H_2_, promoting digestion in the rumen. However, the CH_4_ production is known to result in a loss of dietary energy for the host and contribute to the greenhouse effect. Our results show that the relative abundance of methanogens in the AHHR diet was lower than in the AS diets. This may be due to the few methanogens after ensiling that were still present, as increasing RDS in the AS diets can decrease the diversity of methanogens. After anaerobic fermentation of plant lignocellulosic materials, lactic acid, formate, and acetic acid are produced, and these substrates are conducive to *Methanosaeta* growth. Although the acidic environments after silage reduced *Methanobacteria* relative abundance from 14% to 4%, it was still higher than that of hay (Zhao et al., 2016). The results of *in vitro* rumen studies found that greater 48h gas and methane production were observed in alfalfa silage than in alfalfa hay (Zhang et al., 2024). In addition, previous studies have established that increasing wheat in the diet decreased CH_4_ production (Moate et al., 2017). However, the effects of the above experiments on methanogens remain unknown. Savin et al. (2022) found that a wheat diet reduced rumen pH and H_2_ for CH_4_ generation, resulting in lower CH_4_ production and a relative abundance of *Methanobacteria*. These results may indicate that the AHHR diet has lower methane production than the AS diet due to the relative abundance of methanogens, which has also been correlated with higher levels of methane emissions (Wallace et al., 2015). There was little impact of diet on the methanogen genera. In the present study, the genera *Methanobrevibacter*, *Methanosphaera*, and *Methanoculleus* were the dominant archaea in the four diets and were similarly distributed, which is consistent with a previous study (Thirumalaisamy et al., 2022). *Methanobrevibacter* and *Methanosphaera* belong to Methanobacteria class, which usually account for more than 90% of methanogen 16S rRNA gene reads. *Methanoculleus* belongs to the Methanomicrobia class. *Methanobrevibacter* and *Methanoculleus* perform methanogenesis from CO_2_ with H_2_ and formate, while *Methanosphaera* uses H_2_ and methanol for methanogenesis (Garcia et al., 2000). In addition, we explored the methanogen biomarkers (at the species level) in different diets using LEfSe analysis. *Methanobrevibacter_sp_YE315* and *Methanobrevibacter_sp_AbM4* were abundant in the ASLR diet and *Methanobrevibacter_millerae* was abundant in the ASHR diet. This may be due to differences in rumen fermentation pH and pathways (Savin et al., 2022). Dong et al. (2019) reported that butyrate was positively related to *Methanobrevibacter_sp_AbM4*.

The changes in the fungal community in the rumen have seldom been described in the literature when feeding alfalfa forms or RDS diet, thus, our data fill this gap. Fungi are known to play a key role in the degradation of plant lignocellulosic materials through the production of enzymes (Elekwachi et al., 2017; Gruninger et al., 2014). Fungal counts are usually low before feeding AH, while AS can reach 7.74×107mL^-1^ (Chen et al., 2021). However, we found that the relative abundance of fungi was higher in the AH diets than in the ASHR diet in the rumen, and diversity was higher in the LR diets than in the HR diets. This indicates that the growth of fungi was promoted by more lignin substrates in the AH diets, and inhibited by the low pH or other ensiling products in the AS diets (Shen et al., 2020). Although a high RDS diet usually reduces rumen pH (Li et al., 2014), rumen pH in our same study did not show a difference (Guo et al., 2025a). At the level of fungal phylum, our research found that *Ascomycota* and *Basidiomycota* are the major fungi in the rumen of sheep, in line with our results (Wei et al., 2024; Han et al., 2019). At the genus level of fungi, the current results are not in agreement with previous findings in sheep. Kittelmann et al. (2013) reported that *Neocallimastix* (28%), *Piromyces* (20%), *Orpinomyces* (12%), *BlackRhino* (8%), *Caecomyces* (8%), and *Cyllamyces* (5%) were the predominant anaerobic fungal genera in the rumen. In addition, Han et al. (2019) reported that *Neocallimastix* was the most abundant anaerobic fungal genus in the rumen. However, our findings showed that the predominant fungi detected were from the genera *Candida*, *Sarocladium*, and *Penicillium*, which collectively accounted for more than 50% of the fungal genus reads and have the same function as degrading fibers. *Aspergillus* was the only fungal genus with a higher relative abundance in the sheep fed the AHLR diet compared to the AS diets, indicating that the high fiber and low RDS promoted the proliferation of *Aspergillus*, which are mainly involved in the degradation of lignin (Nidhina et al., 2017). In addition, we explored the fungal biomarkers in the four diets using LEfSe analysis. We found that the different diets significantly affected the rumen fungal community. Specifically, the specific fungal biomarkers in the AHLR diet were *Aspergillus*, *Metschnikowia*, *unclassified_f:Stachybotryaceae*, and *Stachybotrys*; *Stemphylium* and *Cystobasidium* in the AHHR diet; *unclassified_k:Fungi*, *Trichothecium*, and *Psathyrella* in the ASLR diet; and *Alfaria* in the ASHR diet. Among them, the strong fiber degradation function of *Aspergillus* in the rumen has been reported (Nidhina et al., 2017; Tulsani et al., 2022), however, the function of most other fungi in the rumen is still unknown.

The triangular heat map shows the internal relationships among the methanogens. Our findings revealed that the relative abundance of *Methanobrevibacter* was negatively correlated with *Methanosphaera* in all diets, which is due to different methanogenesis pathway competition for H_2_ within the rumen (Li et al., 2024; Garcia et al., 2000). For fungi, *Penicillium*, *Cladosporium*, and *Exophiala* showed positive correlations among themselves. These fungi can degrade carbohydrates to produce formic acid, H_2_, and CO_2_, which in turn promotes the growth of *Methanobrevibacter* in the rumen (Han et al., 2019). Moreover, *Candida* was negatively associated with *Sarocladium*, as both can degrade lignocelluloses (Marrero et al., 2015; Hou et al., 2019). Those with comparable nutrition patterns may compete with each other (Johnston et al., 2019). Additionally, *Aspergillus* had a negative association with *Occultifur*. These results indicate that there is always a strong pattern of association among methanogens and fungi despite perturbations in dietary changes.

We integrated the changes in methanogens and fungi with the phenotypic characteristics previously observed. The methanogen Chao 1 and Shannon indexes had a negative correlation with BCP, suggesting that the proliferation of rumen microbiota may have reduced the relative abundance of low-abundance methanogens (Tian et al., 2023). The fungal Shannon index was negatively correlated with ADG. This again proves that microbiota interactions could be more important to ecosystem functioning than microbiota diversity in high feed efficiency ecosystems (Wang et al., 2023). In addition, *Methanobrevibacter*, *Methanoculleus*, and *Penicillium* exhibited a positive correlation with branched-chain VFAs, which indicates extensive degradation of AS protein and fiber, with amino acids and fibers degrading to form branched-chain VFAs and formic acid, respectively (Guo et al., 2025a; Wang et al., 2024). Meanwhile, the relative abundance of *Methanobacteria* was higher in the AS diets in the current study (Chao 1 index). Formate is not only a substrate that increases *Methanobacteria* growth but also *Penicillium* growth (Wang et al., 2019). *Cladosporium* and *Exophiala* also exhibited a negative correlation with VFAs. Many researchers have indicated that the enzymatic action of anaerobic rumen fungi forms VFAs, along with CO_2_ and H_2_ as fermentation endproducts (Bhagat et al., 2023). Thus, the methanogens and fungi have a certain symbiotic relationship. After the substrate was depleted and the VFAs concentration increased, the growth of fungi was inhibited. Although our study provided new insights into the effects of different alfalfa forms and RDS levels on rumen methanogens and fungi, future research is needed to determine their functional role and interaction relationships using metagenomic sequencing techniques, and such information can provide greater insights into how diets affect performance.

## Conclusion

The present findings showed RDS levels altered rumen richness and diversity of methanogens and fungi in sheep fed AH or AS. The increased level of RDS in the AH diets reduced the methanogen Chao 1 index compared to the AS diets, and the Shannon index was reduced compared to the ASLR diet. The AH diet fungi Chao 1 index was higher than the ASHR diet, and the LR diets*’* Shannon index was higher than the HR diets. Some species and genera were also affected differently by the four dietary treatments. The correlation analysis found some genera (*Methanobrevibacter*, *Methanoculleus*, *Penicillium*, *Cladosporium*, and *Exophiala*) were positively correlated with our previously observed concentrations of isobutyrate and isovalerate, and may provide greater insights into the previously observed differences.

## Data Availability

The raw data supporting the conclusions of this article will be made available by the authors, without undue reservation.
