# Supplementary material for: Relationships between rumen methanogens and fungal communities and their response to changes in alfalfa forms and starch in sheep diets
Source: Front Microbiomes. 2025 Apr 9;4:1567462. doi: 10.3389/frmbi.2025.1567462 (PMC12993492; doi:10.3389/frmbi.2025.1567462)
Supplement: Supplementary file 1 [file Table1.docx]

Additional file

Table S1 Effect of dietary alfalfa forms and RDS levels on growth performance and rumen fermentation parameters in sheep

| **Items** | **Treatments ^1^** | | | | **SEM** | ***P*-Value ^2^** | | | |
| --- | --- | --- | --- | --- | --- | --- | --- | --- | --- |
|  | **AHLR** | **AHHR** | **ASLR** | **ASHR** |  | **Diet** | **Alfalfa forms** | **RDS levels** | **Forage × RDS** |
| Dry matter intake, g/d | 1039.06 | 1121.17 | 980.34 | 1072.27 | 43.51 | 0.132 | 0.205 | 0.045 | 0.907 |
| Average daily gain, g/d | 156.12 ^b^ | 195.88 ^a^ | 166.66 ^ab^ | 203.14 ^a^ | 15.03 | 0.029 | 0.217 | 0.003 | 0.565 |
| F:G | 6.99 ^a^ | 6.06 ^ab^ | 6.11 ^ab^ | 5.29 ^b^ | 0.44 | 0.065 | 0.062 | 0.048 | 0.892 |
| NH_3_-N, mg/100ml | 7.66 ^c^ | 8.37 ^bc^ | 15.39 ^a^ | 10.53 ^b^ | 0.66 | 0.001 | 0.001 | 0.010 | 0.002 |
| TAA, μmol/ml | 16.67 ^a^ | 13.53 ^b^ | 18.03 ^a^ | 16.19 ^a^ | 0.52 | 0.013 | 0.126 | 0.032 | 0.678 |
| BCP, mg/ml | 33.46 ^b^ | 54.24 ^a^ | 28.01 ^b^ | 30.85 ^b^ | 2.71 | 0.001 | 0.003 | 0.009 | 0.055 |
| Acetate | 70.31 | 71.81 | 80.25 | 77.58 | 5.12 | 0.449 | 0.139 | 0.910 | 0.690 |
| Propionate | 29.54 | 27.55 | 28.41 | 25.01 | 3.53 | 0.841 | 0.621 | 0.467 | 0.849 |
| Isobutyrate | 3.01 ^c^ | 3.18 ^bc^ | 4.36 ^a^ | 4.01 ^ab^ | 0.37 | 0.016 | 0.002 | 0.794 | 0.424 |
| Butyrate | 3.47 | 3.28 | 3.92 | 4.18 | 0.43 | 0.460 | 0.133 | 0.929 | 0.617 |
| Isovalerate | 2.17 | 2.29 | 2.96 | 2.42 | 0.34 | 0.984 | 0.187 | 0.535 | 0.342 |
| Valerate | 0.93 | 0.93 | 1.16 | 1.27 | 0.13 | 0.179 | 0.035 | 0.681 | 0.674 |
| TVFA | 109.43 | 109.04 | 121.05 | 114.47 | 8.37 | 0.737 | 0.331 | 0.689 | 0.722 |
| Lactic acid | 0.53 ^a^ | 0.39 ^bc^ | 0.46 ^ab^ | 0.32^c^ | 0.04 | 0.242 | 0.029 | 0.001 | 0.905 |

^1^ AHLR: alfalfa hay and low (14.85% DM) RDS, AHHR: alfalfa hay and high (20.21% DM) RDS, ASLR: alfalfa silage and low (14.85% DM) RDS, ASHR: alfalfa silage and high (20.21% DM) RDS. ^a–b^ Significant differences within a row with different superscripts (*P* < 0.05). *P* (Diet) = four dietary treatments. *P* (Alfalfa) = alfalfa hay versus alfalfa silage (AH vs. AS); *P* (RDS) = low (14.85% DM) RDS versus high (20.21% DM) RDS (LR vs. HR); *P* (Alfalfa×RDS) = alfalfa forms by RDS levels interaction.
